# Fracture Risk Assessment in Atypical Parkinsonian Syndromes

**DOI:** 10.1002/mdc3.13146

**Published:** 2021-01-31

**Authors:** Christopher Kobylecki, Hannah Glasse, Jigisha Amin, Celia L. Gregson, Veronica Lyell, Emily J. Henderson

**Affiliations:** ^1^ Department of Neurology, Manchester Centre for Clinical Neurosciences Salford Royal NHS Foundation Trust Salford United Kingdom; ^2^ Manchester Academic Health Sciences Centre University of Manchester Manchester United Kingdom; ^3^ Faculty of Health Sciences, Bristol Medical School University of Bristol Bristol United Kingdom; ^4^ Musculoskeletal Research Unit, Bristol Medical School University of Bristol Bristol United Kingdom; ^5^ Older People's Unit Royal United Hospitals Bath NHS Foundation Trust Bath United Kingdom; ^6^ Department of Population Health Sciences, Bristol Medical School University of Bristol Bristol United Kingdom

**Keywords:** fracture risk, Parkinson's disease, multiple system atrophy, progressive supranuclear palsy, osteoporosis

## Abstract

**Background:**

Bone health and fracture risk reduction are increasingly recognized as important issues in Parkinson's disease (PD). However, the evidence for fracture risk management in atypical parkinsonism (AP) is less clear. Guidance on management of bone health in PD has recently been published.

**Objectives:**

To evaluate the outcome of fracture risk assessment in a cohort of patients with AP, compared to a population with idiopathic PD.

**Methods:**

We did a cross‐sectional study of patients with PD or AP who had fracture risk assessed at two tertiary movement disorder centres. Data on fracture risk as assessed using QFracture and FRAX were collected. To assess for the effect of age on fracture risk we compared the risks of PD and AP patients aged ≤70 and >70 years.

**Results:**

We assessed 71 patients with AP and 267 with PD. Age, sex and body mass index were similar between groups; patients with AP were more likely to have fallen in the previous year. Major osteoporotic fracture risk was greater in patients with AP aged ≤70 compared to PD; no differences between groups were seen in those aged >70 years. 76% of those with AP, and 63% with PD, had an estimated fracture risk indicating bone‐sparing treatment, but only 33% of patients with AP were receiving this where it was indicated.

**Conclusion:**

There is scope for considerable improvement in fracture risk assessment and treatment in atypical parkinsonism, taking into account the worse prognosis of this patient group.

The importance of considering fracture risk and maintaining bone health is an increasingly recognized issue in people with Parkinson's disease (PD). People with PD have a greater than two‐fold risk of osteoporotic fracture than age‐matched healthy controls; this is particularly so in men with PD who have an almost three‐fold increased risk.^1^, ^2^ Multiple factors contribute to this increased risk, including increased risk of falling, vitamin D deficiency, reduced physical activity and impaired nutrition.^3^ Atypical parkinsonian syndromes such as multiple system atrophy (MSA) and progressive supranuclear palsy (PSP) are more rapidly progressive than PD, exhibit a poor response to dopaminergic therapy and are associated with a reduced latency to falling and a high incidence of fractures.^4^

Given the potential for increased risk of fractures in patients with atypical parkinsonism (AP), there is relatively little supporting literature or disease‐specific evidence base regarding management of bone health in these conditions. Whilst one prospective cohort study failed to identify an increased risk of fractures in AP compared to PD, these findings may have been explained by the reduced mobility and therefore reduced risk of falling that may occur with advanced AP.^5^ Yarnall and colleagues identified previous fractures in one quarter of their outpatient clinic population (29 PSP, 7 MSA), despite which only two of these patients were currently on bone protection.^6^ Fracture risk appears to be an under‐appreciated issue in patients with AP.

Since these earlier papers, guidance on assessment and treatment of fracture risk in parkinsonian syndromes has been published.^3^ This guidance suggested the use of QFracture^7^ to assess 10‐year risk of major osteoporotic fracture, with a threshold of major osteoporotic fracture (MOF) risk ≥20% and/or neck of femur fracture (NOF) risk ≥5% to trigger empirical bone protection treatment.^3^ Our aim was to evaluate the outcome of fracture risk assessment and subsequent management in patients with AP as part of a clinical service review, and compare findings to a population of patients with idiopathic PD.

## Methods

We conducted a cross‐sectional study of patients with atypical parkinsonism and idiopathic PD who were assessed at one of two specialist movement disorder clinics in Salford Royal NHS Foundation Trust and Royal United Hospitals Bath NHS Trust by specialists in movement disorders and/ or bone health. We included all patients with these diagnoses assessed between December 2015–December 2016. Demographic data and information on risk factors for fracture were collected directly from medical records into a custom‐built database (Microsoft Excel). Information on treatments for bone health (calcium, vitamin D, bisphosphonates, denosumab) at the time or immediately following the consultation were also recorded. Patients were weighed and height measured; BMI was calculated as weight (kg)/height (m^2^). The 10 year risks of hip and major osteoporotic fracture were calculated using QFracture^7^ and FRAX.^8^

Continuous data were compared between PD and AP using the student's t test if normally distributed or the Mann–Whitney U test if not normally distributed. Categorical variables were compared using the Chi‐squared test. To determine to what extent fracture risk differed by age, we stratified our study population as >70 or ≤70 years, using this cutoff as patients under 70 are often considered “young” in a movement disorders setting and may be presumed to be at lower risk of fracture. Statistical analysis was undertaken using SPSS 23.0 (IBM).

## Results

We analyzed a total of 71 patients with AP (21 MSA, 31 PSP, 12 corticobasal syndrome, 7 dementia with Lewy Bodies) and 267 with idiopathic PD. Demographic details are shown in Table 1. The AP and PD groups were similar in terms of age, sex and BMI. The proportion of patients reporting a prior fracture or a parental history of fracture did not differ between groups. Patients with AP were more likely to have fallen within the last year, than patients with PD (53/71 [74.6%] vs. 110/267 [41.1%], *P* < 0.001). Full information on components of QFracture and FRAX assessments is provided in supplementary Table S1.

**TABLE 1:** Risk factors, treatments and calculated fracture risks for patients with Parkinson's disease and atypical Parkinsonism

			Atypical Parkinsonism groups			
	PD n = 267	Atypical Parkinsonism n = 71	MSA n = 21	PSP n = 31	CBS n = 12	DLB n = 7
Age	75.4 (8.9)	73.1 (7.9)	69.1 (8.6)	74.9 (7.2)	73.3 (7.7)	77.4 (4.9)
Sex	104 F 163 M	28 F 43 M	7 F 14 M	13 F 18 M	7 F 5 M	1 F 6 M
BMI	26.2 (5.8)	25.7 (6.0)	25.6 (8.0)	24.9 (6.0)	24.9 (3.9)	28.5 (4.1)
Previous fragility fracture (%)	47 (17.6)	13 (18.3)	5 (23.8)	6 (19.3)	2 (16.7)	0 (0)
Parental hip fracture (%)	21 (7.9)	9 (12.7)	4 (19.0)	5 (16.1)	3 (25)	0 (0)
Fall in the last year (%)	110 (41.1)	53 (74.6)***	17 (81.0)	26 (83.9)	5 (41.7)	5 (71.4)
Vitamin D supplement (%)	135 (50.6)	41 (57.7)	13 (61.9)	16 (51.6)	7 (58.3)	5 (71.4)
Calcium supplement (%)	102 (38.2)	40 (56.3)**	13 (61.9)	15 (48.3)	7 (58.3)	5 (71.4)
Prescribed oral bisphosphonate (%)	42 (15.7)	18 (25.4)	3 (14.2)	9 (29.0)	4 (33.3)	2 (28.6)

PD, idiopathic PD; MSA, multiple system atrophy; PSP, progressive supranuclear palsy; CBS, corticobasal syndrome; DLB, dementia with Lewy Bodies. BMI, body mass index. Data presented as mean (SD), number (%) or median (IQR).

** *P* < 0.01,

*** *P* < 0.001 compared to PD (Chi‐squared test).

**FIG. 1:**
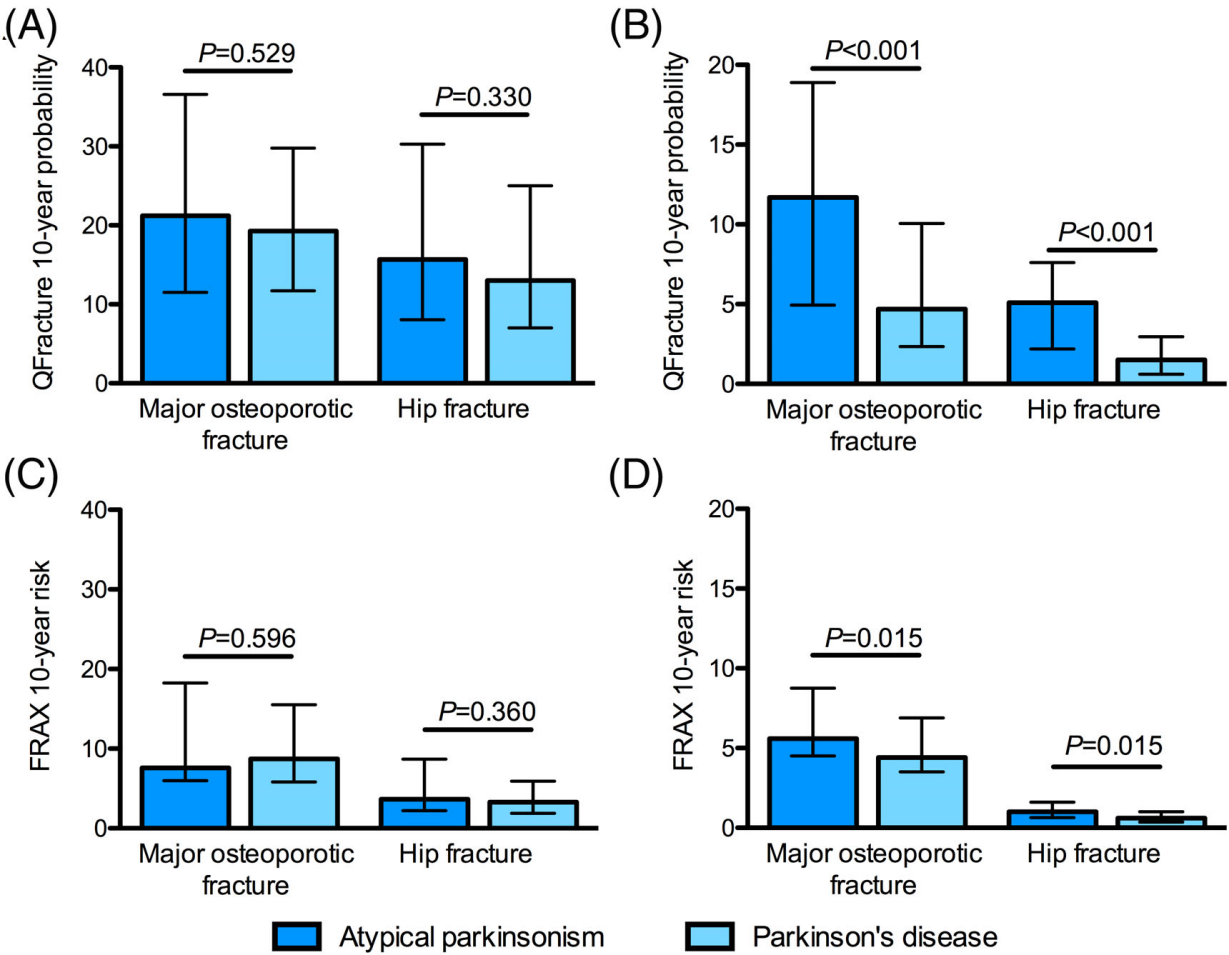
Fracture risks in atypical parkinsonian syndromes and idiopathic Parkinson's disease as assessed by QFracture in those aged >70 (**A**) and ≤70 years (**B**). FRAX assessement is shown in those aged >70 (**C**) and ≤70 years (**D**). Bars represent median 10‐year fracture risk ± interquartile range. NOF, neck of femur. Statistical comparisons made using Mann–Whitney U test.

**FIG. 2:**
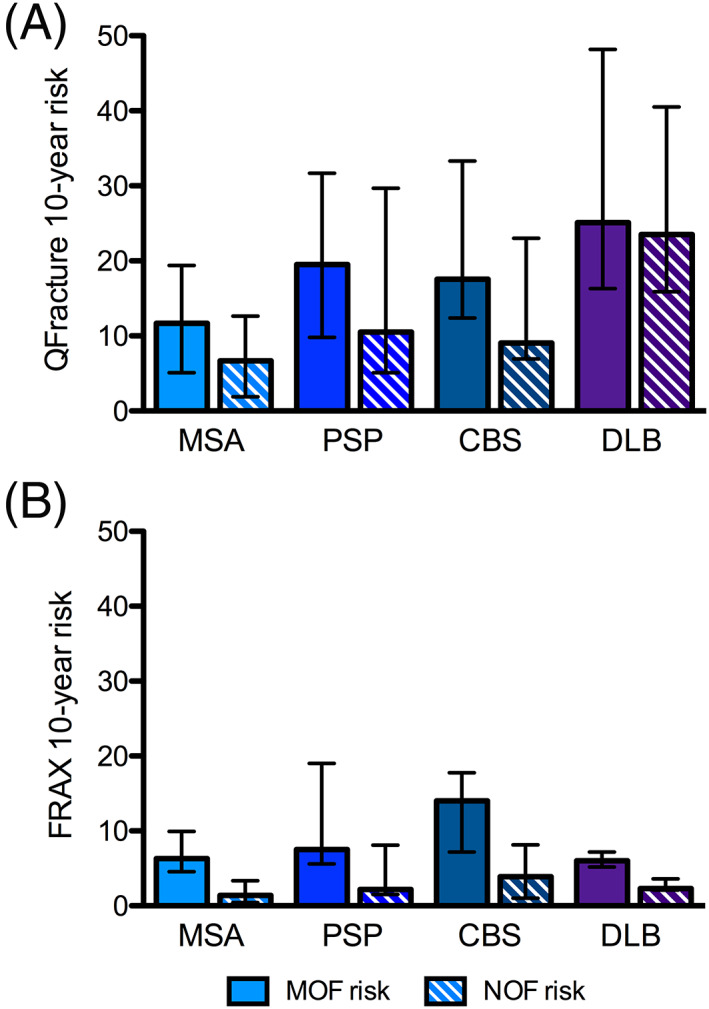
Fracture risks in individual atypical parkinsonian syndromes assessed by QFracture (**A**) and FRAX (**B**). Bars represent median 10‐year fracture risk ± interquartile range. MOF, major osteoporotic fracture; NOF, neck of femur; MSA, multiple system atrophy; PSP, progressive supranuclear palsy; CBS, corticobasal syndrome; DLB, dementia with Lewy bodies.

The 10‐year risks of major osteoporotic fracture (MOF), as calculated by QFracture and FRAX, were similar in the total population of patients with AP and PD and those aged >70 years (Fig. 1A,C). However, when we examined patients ≤70 years old, major osteoporotic fracture risk assessed using QFracture was greater in those with AP (n = 25, median 11.7% [IQR 5–19]) compared to those with PD (n = 73, median 4.7% [2‐10], *P* < 0.001). The risk of hip fracture was also three‐fold higher in patients with AP aged ≤70 years old (median 5.1% [2–8]) compared to those with PD (median 1.5% [1–3], *P* < 0.001; Fig. 1B). When FRAX was used to calculate the 10‐year probability of hip and major osteoporotic fractures, similar patterns were seen (Fig. 1D). There were no differences in sex or BMI between patients with AP and PD in either age group, although falls remained more prevalent in AP in both age categories. Fracture risks for individual atypical parkinsonian syndromes are shown in Fig. 2. There were no significant differences in fracture risk when comparing individual AP syndromes to others.

A total of 54 patients with AP (76%), and 167 (63%) patients with PD had a QFracture MOF risk ≥20% and/or hip fracture risk ≥5%, fulfilling criteria for bone‐sparing treatment. Despite these risks, no differences were seen in the proportion of patients receiving vitamin D or calcium supplementation or oral bisphosphonate therapy, by fracture risk. Only 18 (33%) patients with AP, in whom bisphosphonates were potentially indicated, were receiving this therapy, whereas 35 (65%) were taking vitamin D and calcium supplements. One patient with PD and one with AP received parenteral bisphosphonate therapy; one patient with PD received denosumab therapy. Bone densitometry had been performed in 7 of the AP group (10%) and 39 (15%) of PD patients.

## Discussion

To our knowledge this is the largest report of clinical fracture risk assessment and bone health treatment in patients with atypical parkinsonism. Comparing the risk factors for future fracture between groups, our data indicate that previous fractures had occurred in 18% of both our AP and PD populations. The main difference between the two populations, was the much higher incidence of falls in patients with AP, consistent with the previous literature.^4^ Whereas there were no differences in estimated fracture risk overall, both major osteoporotic fracture and hip fracture risks were higher in younger (aged ≤70 years) patients with atypical parkinsonian syndromes compared to those with PD, which reinforces the importance of evaluating fracture risk in a comparatively younger population. The proportionately greater contribution of advancing age to fracture risk in the older group is likely to explain this discrepancy, whereas a higher level of other risks such as falls in the younger AP group is likely to drive the greater risk seen in this group. Greater differences are seen in assessment of MOF compared to hip fracture,^9^ consistent with our data.

PD and AP are established risk factors for fragility fracture. In 2017, the UK National Institute for Health and Care Excellence clearly stated that fracture risk assessment should be considered in all men and women with a risk factor for fracture.^10^ Thus, all men and women with PD and AP in the UK should routinely be assessed for fracture risk.^10^ Recent guidance on fracture risk assessment specifically in parkinsonism suggests the use of QFracture in life‐limiting conditions when prognosis is <10 years, as opposed to a recommendation to use FRAX in PD assessment where quantification of 10‐year fracture risk has greater clinical utility.^11^ Given the evidence of more rapid progression in MSA and PSP compared to PD, with a mean life expectancy from diagnosis of 7–8 years,^12^ this would support the use of QFracture in the first instance. QFracture as a tool is able to calculate risk over shorter time periods (from 1 to 10 years at one yearly increments) and incorporates more risk factors in the calculation including whether or not the patient has PD and experienced a fall. Falls have been identified as a risk for fractures independently of FRAX‐derived fracture probabilities,^13^ which emphasizes their importance in patients with atypical parkinsonism, where falls occur more frequently. The inclusion of falls in the QFracture algorithm is a potential strength in this regard; FRAX calculations make no distinction on the basis of falls.

Despite appropriate fracture risk assessment in our population with AP, the proportion treated with bone‐sparing therapy was lower than expected. The reasons for this could include poor tolerability and adherence to oral bisphosphonate therapy, as well as a lack of awareness of current guidance by clinicians.^11^ Dysphagia may well contribute to poor adherence given the difficulties with swallowing oral bisphosphonates, highlighting the potential role of parenteral bone‐sparing therapies in this population. However, our data and clinical experience suggests that parenteral treatments seem under utilized. This may be due to the lack of familiarity with using parenteral drugs, concern about potential side effects, access to services, and under‐recognition of fall and fracture risk in this population.

We acknowledge limitations to our work, including the fact that risk assessment may not be representative of generalist clinics without a special interest in bone health, and the lack of follow‐up data to determine long‐term adherence or outcomes from bone‐sparing therapy. Specifically, we do not have data on the rates of further falls or fractures in this population, and the study is not powered to detect the effects of such interventions. Further prospective work to assess real‐world fracture risk following assessment, and the efficacy of bone‐sparing measures in such populations would be valuable. In particular, fracture risk may differ in subtypes of PSP such as PSP‐parkinsonism, given the longer latency to falls in this condition ^4^ as well as between the different AP conditions. Bone‐sparing therapy may not be appropriate when the last year of life is reached, and our data do not explicitly identify those where treatment was considered and then appropriately withheld. It is possible that more widespread bone densitometry measurement would have yielded more precise evaluation of fracture risk, although such measurement is not currently recommended in all parkinsonian patients.^11^ Finally, we cannot identify the number in our population who went on to have further fractures, given that predicted fracture risk may not equate to real‐life risk. Strengths of our study include recruitment from two specialist centres with expertise in movement disorders and bone health, a standardized data collection method and a comparative group of patients with PD.

In conclusion, there remains substantial scope for improvement in ascertainment of fracture risk and incidence and the implementation of evidence‐based therapy to reduce fracture risk in patients with atypical parkinsonism. Current guidance for the use of QFracture in this patient group with fracture risk calculated for a shorter (eg 5 year) time period would accommodate reduced life expectancy whilst identifying those at risk of the serious and life‐threatening complication of osteoporotic fracture.^11^ Widespread, routine roll‐out of this method would help to improve fracture risk assessment in people with atypical parkinsonian syndromes and inform shared‐decision making in risk management for their remaining years of life.

## Author Roles

(1) Research Project: A. Conception, B. Organization, C. Execution; (2) Statistical Analysis: A. Design, B. Execution, C. Review and Critique; (3) Manuscript Preparation: A. Writing of the first draft, B. Review and Critique.

C.K.: 1A, 1B, 2A, 2B, 2C, 3A, 3B

H.G.: 1B, 1C, 2C, 3B

J.A.: 1B, 1C, 3B

C.L.G.: 1A, 1B, 2C, 3B

V.L.: 1A, 1B, 1C, 3B

E.J.H.: 1A, 1B, 2C, 3B

## Disclosures

### Ethical Compliance Statement

As this was a quality improvement project, we confirm that approval of an institutional review board was not required for this work and informed patient consent was not required. We confirm that we have read the Journal's position on issues involved in ethical publication and affirm that this work is consistent with those guidelines.

### Funding Sources and Conflicts of Interest

No specific funding was received for this work, and the authors declare no conflict of interest.

### Financial Disclosures for the Previous 12 Months

Christopher Kobylecki has received grants from Parkinson's UK and the Michael J Fox Foundation; speaker fees from Britannia and Bial Pharma; support to attend international meetings from Abbvie. Hannah Glasse, Jigisha Amin, Celia L Gregson, report no financial disclosures. Veronica Lyell has received fees for work on educational material from Parkinson's UK. Emily J Henderson has received research funding from the National Institute of Health Research (NIHR), Parkinson's‐UK, the Gatsby Foundation. She has received travel, consultancy, and honoraria from Profile Pharma, Bial, Abbvie, and Ever pharma.

## Supporting information


**Table S1**. Additional features contributing to fracture risk assessment in patients with Parkinson's disease and atypical parkinsonism as assessed by QFracture and FRAX.
