# Supplementary material for: Fracture Risk Assessment in Atypical Parkinsonian Syndromes
Source: Mov Disord Clin Pract. 2021 Jan 31;8(3):385–9. doi: 10.1002/mdc3.13146 (PMC8015890; doi:10.1002/mdc3.13146)
Supplement: Supplementary file 1 — Table S1. Additional features contributing to fracture risk assessment in patients with Parkinson's disease and atypical parkinsonism as assessed by QFracture and FRAX. [file MDC3-8-385-s001.docx]

|  | **Parkinson’s disease** | **Atypical parkinsonism** |
| --- | --- | --- |
| Ethnicity | Bangladeshi 2  Chinese 3  Indian 2  Not stated 4  Other Asian 1  Other ethnic group 1  Pakistani 2  White 253 | Pakistani 2  Not stated 1  White 68 |
| Smoking status | Ex-smoker 35  Heavy smoker 1  Light smoker 5  Moderate smoker 4  Non-smoker 222 | Ex-smoker 17  Light smoker 2  Non-smoker 52 |
| Diabetes | Type 1 1  Type 2 29 | Type 2 9 |
| Care home resident | Yes 13  No 254 | Yes 7  No 64 |
| Dementia | 35 | 18 |
| Previous cancer | 29 | 5 |
| Asthma or COPD | 27 | 1 |
| Ischaemic heart disease or stroke | 55 | 7 |
| Chronic liver disease | 0 | 0 |
| Chronic kidney disease | 2 | 0 |
| Rheumatoid arthritis | 2 | 0 |
| Malabsorption | 11 | 2 |
| Endocrine problems | 8 | 1 |
| Epilepsy or taking anticonvulsants | 7 | 3 |
| Taking antidepressants | 72 | 23 |
| Taking steroids | 8 | 2 |
| Oestrogen only HRT | 1 | 0 |
| Alcohol 3 or more units/day | 14 | 1 |

**Supplementary table 1.** Additional features contributing to fracture risk assessment in patients with Parkinson’s disease and atypical parkinsonism as assessed by QFracture and FRAX.
